# eIF3a improve cisplatin sensitivity in ovarian cancer by regulating XPC and p27^Kip1^ translation

**DOI:** 10.18632/oncotarget.4555

**Published:** 2015-07-11

**Authors:** Yu Zhang, Jing-Jing Yu, Yan Tian, Zheng-Zheng Li, Cai-Yi Zhang, Shu-Fen Zhang, Lan-Qin Cao, Yi Zhang, Chen-Yue Qian, Wei Zhang, Hong-Hao Zhou, Ji-Ye Yin, Zhao-Qian Liu

**Affiliations:** ^1^ Department of Clinical Pharmacology, Xiangya Hospital, Central South University, Changsha 410008, P. R. China; ^2^ Institute of Clinical Pharmacology, Central South University, Hunan Key Laboratory of Pharmacogenetics, Changsha 410078, P. R. China; ^3^ Department of Obstetrics & Gynecology, Xiangya Hospital, Central South University, Changsha 410008, P. R. China

**Keywords:** eIF3a, platinum, ovarian cancer, drug resistance, XPC

## Abstract

The eukaryotic translation initiation factor 3a (eIF3a) is one of the core subunits of the translation initiation complex eIF3, responsible for ribosomal subunit joining and mRNA recruitment to the ribosome. Our previous study identified that it was correlated with platinum response in lung cancer. The current study aims to test the hypothesis that eIF3a may affect the drug response and prognosis of ovarian cancer patients receiving platinum-based chemotherapy by regulating xeroderma pigmentosum complementation group C (XPC) and p27^Kip1^. Immunohistochemistry and western blot was used to determine the expression of eIF3a in 126 human ovarian cancer tissues followed by association analysis of eIF3a expression with patient's response and survival. Ectopic over-expression and RNA interference knockdown of eIF3a were carried out in A2780/cisplatin (DDP) and its parental A2780 cells, respectively, to determine the effect of altered eIF3a expression on cellular response to cisplatin by employing MTT assay. Western Blot analyses were also carried out to determine the regulation of eIF3a on XPC and p27^Kip1^. eIF3a expression was associated with response of ovarian cancer patients to DDP-based chemotherapy and their survival. Overexpression and knockdown of eIF3a increased and decreased the cellular response to cisplatin in A2780/DDP and A2780 cells, respectively. In addition, XPC and p27^Kip1^ were down regulated by eIF3a. eIF3a improves ovarian cancer patients' response to DDP-based chemotherapy via down regulating XPC and p27^Kip1^.

## INTRODUCTION

Epithelial ovarian cancer (EOC) is one of the most common gynecologic malignancies, and the fifth most frequent cause of death by cancer in women in the United States [1]. In China, EOC is also one of the top 10 most commonly cancers in the female population. In 2011, the total ovarian cancer cases in China were 45,233 and the incidence was 6.89/10^5^, which account for 3.11% for all female cancer patients [2]. The patients are commonly diagnosed lately with advanced disease. In spite of high response rates to the standard first-line treatment for advanced disease with primary debulking surgery, followed by cisplatin (DDP)-based chemotherapy, more than 70% of the patients eventually relapse developing drug-resistant disease [3]. In addition, although patients may respond firstly to the therapy, the cancer often becomes resistant to further chemotherapy, at this point, the number of effective treatment options is limited. Therefore, for EOC, clinically useful markers that identify DDP resistant tumors among the overall high number of chemosensitive patients, remains a critical need. If identified early, DDP resistant EOC patients could benefit from alternate and/or additional therapeutic options in first-line therapy. Moreover, reliable early identification of DDP resistance may allow the development of clinical trials specifically targeting this population with novel alternate therapies.

The eukaryotic translation initiation factor 3a (eIF3a) is one of the core subunits of the translation initiation complex eIF3, responsible for ribosomal subunit joining and mRNA recruitment to the ribosome [4]. It is known to play an important role in translation initiation as well as in the regulation of various gene products, including tubulin, ribonucleotide reductase M2 and some DNA repair molecules [5–7]. These proteins influence cell growth, proliferation [5], cell cycle [8], differentiation [9], cancer progression and the DNA repair pathways [10, 11]. In our previous study, eIF3a up-regulation was identified to be correlated with better prognosis and response to DDP-based chemotherapy in lung cancer patients [12, 13]. Our *in vitro* study also showed that eIF3a knockdown or overexpression, respectively, increased and decreased the cellular resistance to DDP and anthrocycline anticancer drugs [12, 14]. Furthermore, some *eIF3a* polymorphisms were potentially considered as tools for diagnosis and pretreatment evaluation of DDP-based chemotherapy in breast [15] and lung cancer [16, 17]. Therefore, eIF3a is emerging as a regulator and potential DDP-based chemotherapy response marker.

The xeroderma pigmentosum complementation group C (XPC) protein plays an important role in the nucleotide excision repair (NER) pathway. It is involved in the recognition and initiation of the NER, which plays an important role in removing damaged genes, maintaining the genomic integrity and preventing carcinogenesis. It was reported that the regulation of XPC expression is associated with platinum response. p27^kip1^ is an important inhibitory protein of cyclin dependent kinases (CDKs), it plays a fundamental role in cell cycle progression. The regulation of p27^kip1^ expression occurs at different levels and it was reported that eIF3a negatively regulating translation of p27^kip1^. Thus, we investigated the role of these two proteins in eIF3a regulating ovarian cancer platinum response.

In this study, we explored the role of eIF3a in DDP response in ovarian cancer treatments. We found that eIF3a expression correlated with response of ovarian cancer patients to DDP-based chemotherapy. Its knockdown or ectopic overexpression, respectively, increased and decreased the cellular resistance to cisplatin.

## RESULTS

### eIF3a expression correlates with platinum chemotherapy response

**Figure 1:**
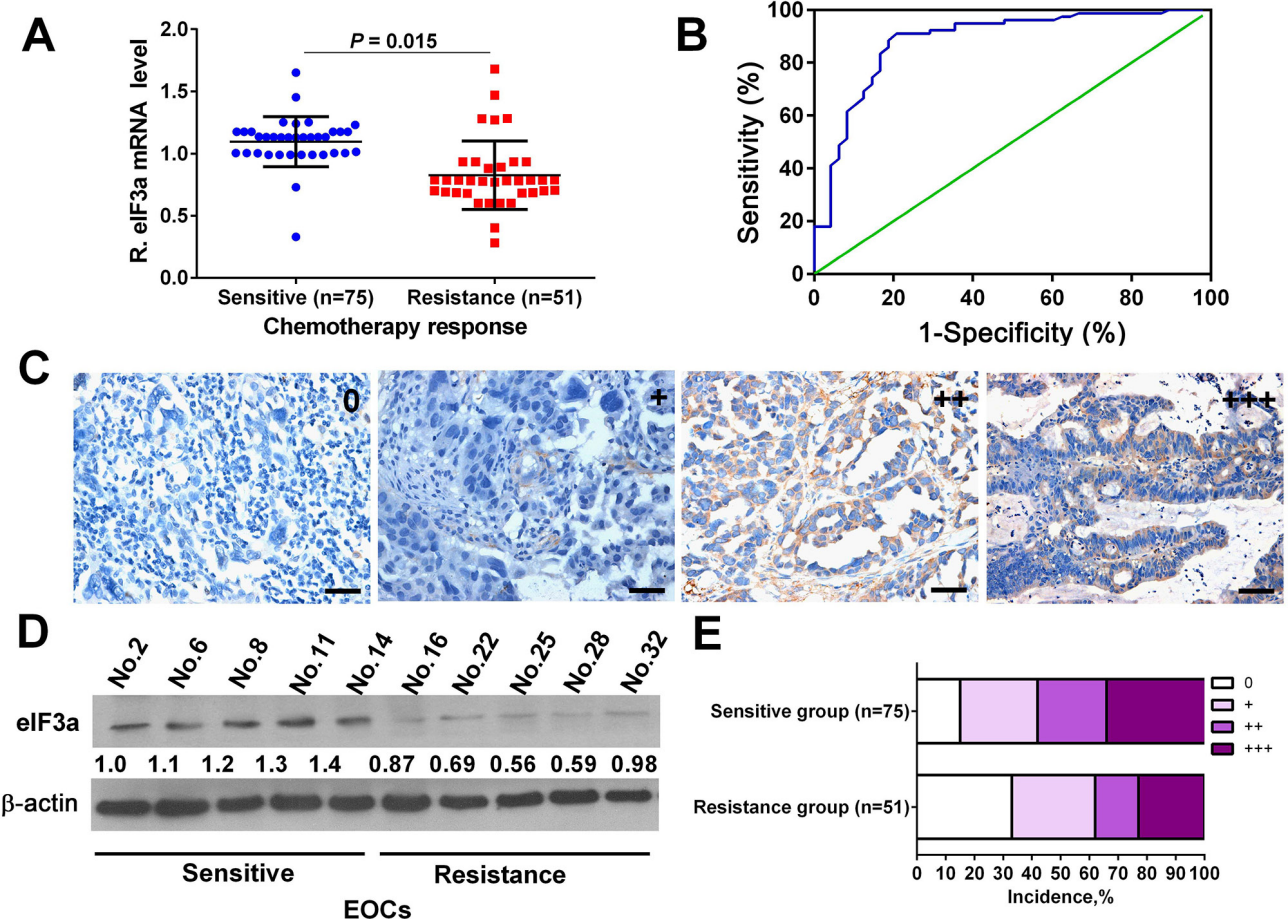
eIF3a expression in human ovarian tumor tissues of DDP sensitive and DDP resistance patients **A.** and **D.** The mRNA and protein levels of eIF3a in DDP sensitive specimens were higher than that in DDP-resistance specimens; **B.** The ROC curve for determining cutoff score for eIF3a expression; **C.** Representative IHC staining of eIF3a expression in the human ovarian tumor tissues; **E.** Percentage distribution of different eIF3a expression patients in the DDP sensitive and resistant specimens.

The eIF3a mRNA and protein expression level in cancer tissues of these patients were firstly evaluated by realtime reverse transcriptase (RT)-PCR (Figure 1A) and immunohistochemistry (IHC) (Figure 1B and 1C), respectively. To confirm the results of IHC, we further analyzed eIF3a expression by Western Blot in some randomly selected samples, as indicated in Figure 1D, the results of both detection methods were consistent with each other. Then, we explored the association of eIF3a expression with chemotherapy response, the results were summarized in Table 1. eIF3a staining did not appear to have any significant correlation with the age and histologic type listed in the table. However, eIF3a staining had significant correlation with chemotherapy responses of DDP (*P* = 0.002). In general, chemotherapy sensitive patients had higher eIF3a expression. Approximately 59.5% of DDP-sensitive EOC patients had high eIF3a level, these numbers go down to 40.5% for DDP-resistance EOC patients (Figure 1E). Thus, the level of eIF3a expression correlates with chemotherapy responses of EOC patients and the increased eIF3a expression may increase chemosensitivity.

**Table 1:** Correlation between eIF3a expression and clinicopathological characteristics in ECO patients

Clinical and pathological variables	eIF3a expression level*		*P*
	Low (%)	High (%)	
Age, years			
≤60	28 (35.44)	51 (64.56)	
>60	17 (36.17)	30 (63.83)	0.934
Chemotherapy response			
Sensitive	35 (46.67)	40 (53.55)	
Resistance	10 (19.61)	41 (80.39)	0.002
Histologic type			
Serous	25 (41.67)	35 (58.33)	
Mucinous	11 (33.33)	22 (66.67)	
Endometrioid	6 (30.00)	14 (70.00)	
Clear cell	3 (23.08)	10 (76.92)	0.533
Pathological grade			
I	13 (68.42)	6 (31.58)	
II	12 (32.43)	25 (67.57)	
III	20 (28.57)	50 (71.43)	0.005
FIGO stage			
I–II	26 (54.17)	22 (45.83)	
III–IV	19 (24.36)	59 (75.64)	0.002

Note: EOC: epithelial ovarian cancer.

* Receive operating characteristic (ROC) curve analysis was employed to assess cut-off score for expression level.

### Higher eIF3a expression patients have better prognosis

**Figure 2:**
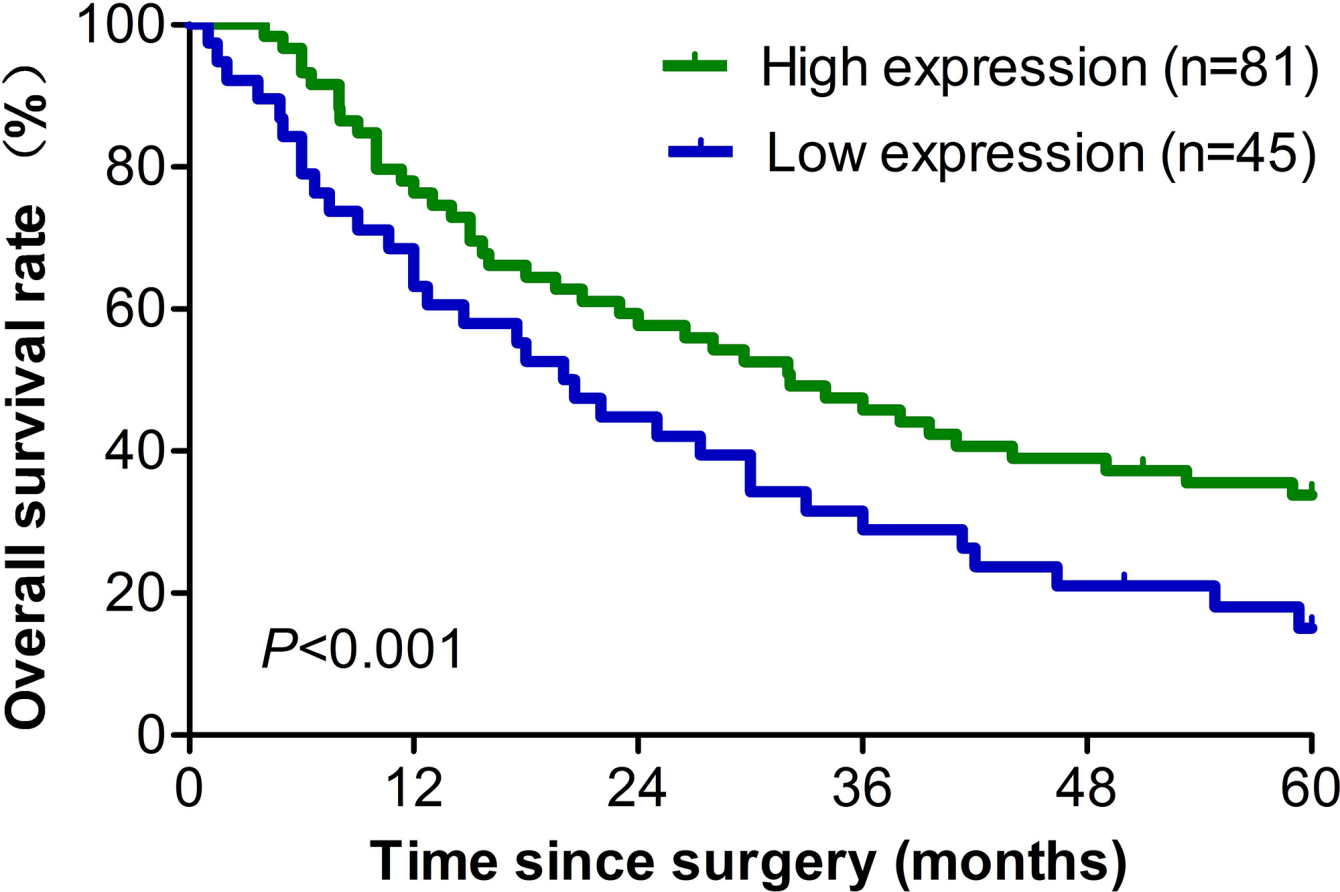
Survival analyses of 126 ovarian cancer patients with high and low eIF3a expression level All patients received DDP based chemotherapy, high eIF3a expression group had longer overall survival than patients in the low eIF3a expression group.

To further investigate the relationship between eIF3a expression and EOC prognosis, we conducted the survival analysis for these patients. As indicated in Table 2, In multivariable Cox regression analysis, eIF3a expression was a significant independent factor that correlated with the overall survival (OS) and relapse-free survival (RFS) of EOC patients (*P* < 0.001 for both). In addition, the postoperative 1, 3, and 5 year OS rates of EOC patients with eIF3a high expression group were significantly higher than those with eIF3a low expression (Figure 2).

**Table 2:** Univariate and multivariate Cox regression analysis of eIF3a expression with survival in EOC patients

Variables	OS				RFS			
	Univariate HR (95% CI)	*P* value	Multivariate HR (95% CI)	*P* value	Univariate HR (95% CI)	*P* value	Multivariate HR (95% CI)	*P* value
Age, years (>60 *vs*. ≤60)	1.05 (0.83–1.26)	0.432	NA	NA	1.03 (0.68–1.40)	0.576	NA	NA
Histological type (serous *vs*. non-serous)	1.136 (0.94–1.38)	0.096	NA	NA	1.10 (0.88–1.56)	0.127	NA	NA
Chemotherapy response (sensitive *vs*. resistance)	2.15 (1.38–3.87)	0.003	1.84 (1.16–2.92)	0.008	1.66 (1.10–2.58)	0.010	2.46 (1.59–4.83)	<0.001
Pathological grade (grade III *vs*. grade II *vs*. grade I)	1.23 (1.16–1.90)	0.021	1.48 (1.28–2.45)	0.002	1.48 (1.18–1.98)	0.018	1.27 (1.11–1.68)	0.024
FIGO stage (III–IV *vs*.I–II)	1.89 (1.28–3.57)	0.009	2.02 (1.37–4.22)	<0.001	2.03 (1.23–3.83)	0.003	2.32 (1.41–4.19)	0.001
Level of eIF3a expression (high *vs*. low)	2.67 (1.45–4.24)	<0.001	3.88 (1.76–6.31)	<0.001	3.07 (1.53–5.44)	<0.001	3.37 (1.86–5.94)	<0.001

Note: EOC, epithelial ovarian cancer. NA, not available.

### eIF3a increase DDP sensitive in ovarian cancer cells

**Figure 3:**
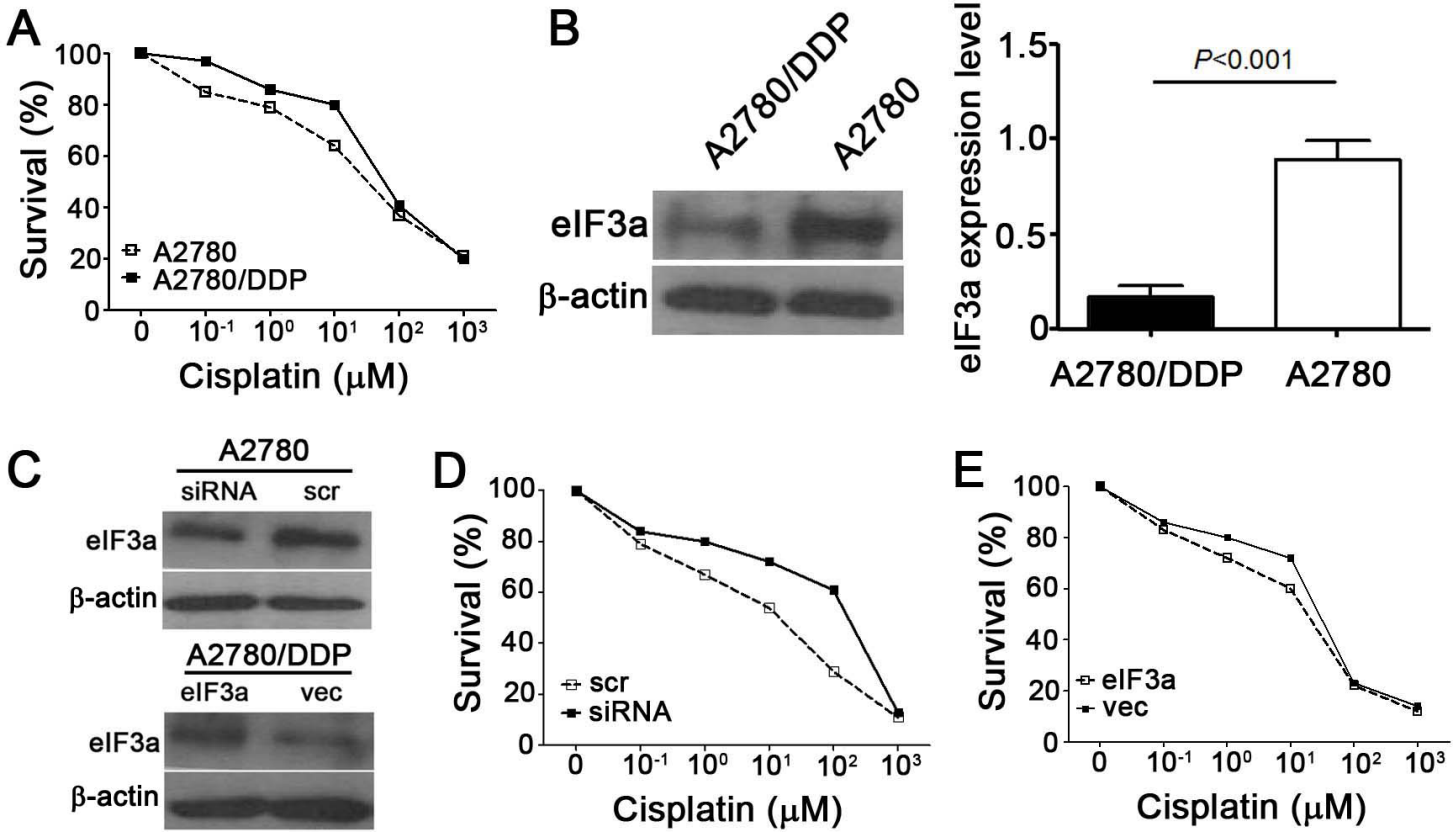
Effect of eIF3a on cellular response to DDP **A.** A2780/DDP were more resistant to DDP compared with its parental A2780 cells. Both cells were treated with various concentrations of DDP for 72 hours and followed by MTT assay; **B.** eIF3a expression was decreased in the A2780/DDP cells; **C.** Knockdown and overexpression of eIF3a in A2780 and A2780/DDP cells, respectively; **D.** A2780 cells with eIF3a knocked down were less sensitive to cisplatin compared to the cells transfected with scramble control; **E.** A2780/DDP cells with eIF3a over-expression were more sensitive to cisplatin compared to the cells transfected with vector control. All proteins were detected using western blot, and relative expression level was determined using gel densitometer. Actin was used as loading control. The data were from 3–6 independent experiments. scr: scramble control, vec: vector control.

Based on our previous results that eIF3a expression correlated with DDP resistant in lung cancer, we next tested whether eIF3a also affected DDP response in EOC cell lines. The parental A2780 and its DDP resistant derivative A2780/DDP cells were used in the current study, as shown in Figure 3A, the A2780/DDP cells were obviously more resistant to DDP. And the eIF3a protein level in A2780 was significantly higher than that in A2780/DDP cells (Figure 3B), indicating that eIF3a may be correlated with DDP resistance. Then, we conducted the experiments of knockdown and overexpression of eIF3a in A2780 and A2780/DDP cells, respectively. As indicated in Figure 3C, eIF3a expression was successfully reduced by siRNA and up-regulated by eIF3a open reading frame (ORF) clone transfection, as determined by using western blot analyses. These cells were then subjected to methyl thiazolyl tetrazolium (MTT) assay, the results showed that A2780 cells with down-regulated eIF3a were more resistant to DDP (Figure 3D). In contrary, the A2780/DDP cells with eIF3a over-expression were more sensitive to DDP compared to the control cells transfected with vector control (Figure 3E). Thus, we concluded that overexpression of eIF3a increased DDP response in the EOC cells.

### eIF3a regulated p27^Kip1^ and XPC expression

**Figure 4:**
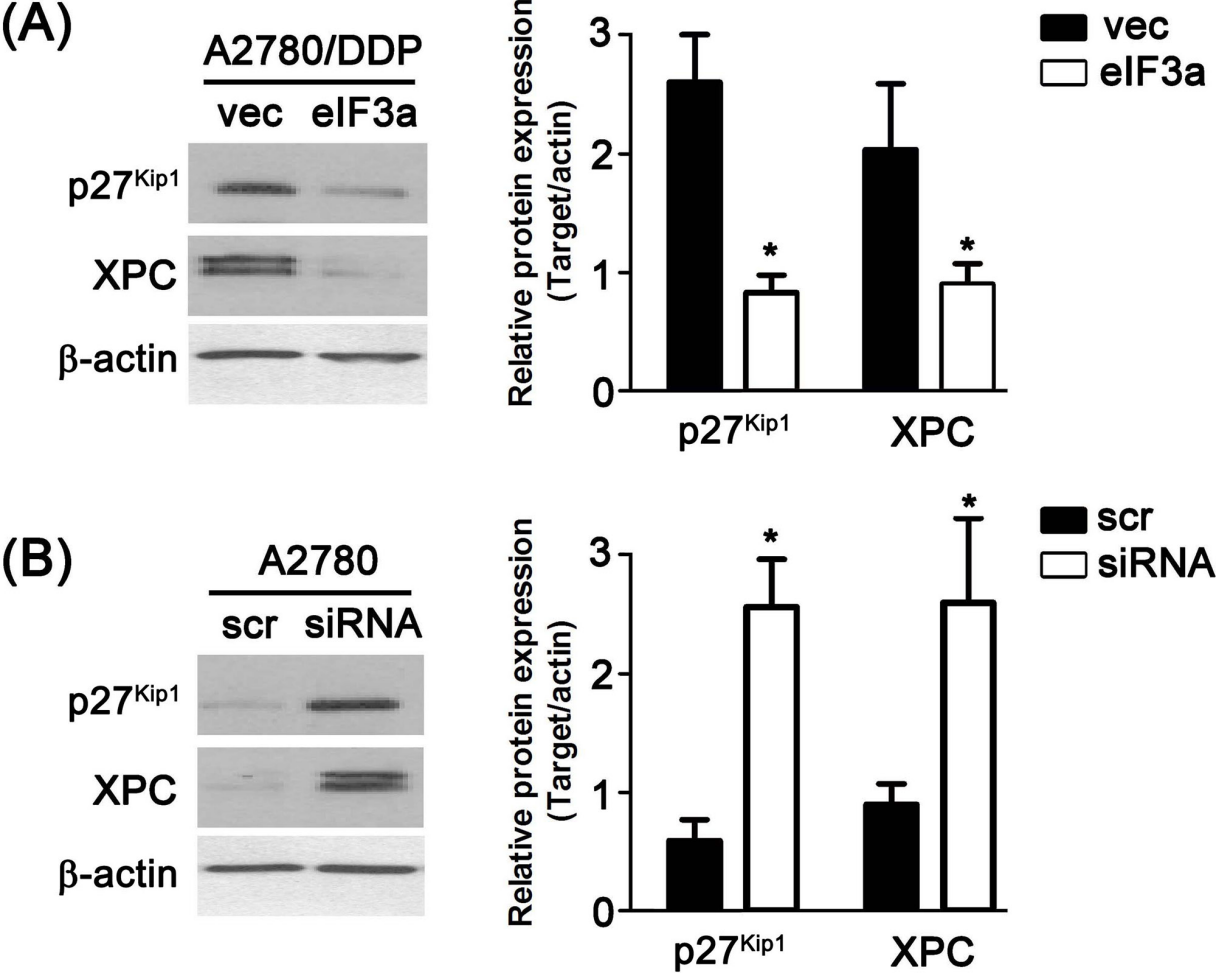
Regulation of eIF3a to XPC and p27^Kip1^ **A.** Over expression of eIF3a down regulated the protein level of XPC and p27^Kip1^ in A2780/DDP cells; **B.** Knockdown of eIF3a up regulated the protein level of XPC and p27^Kip1^ in A2780 cells. All proteins were detected using western blot, and relative expression level was determined using gel densitometer. Actin was used as loading control. The data were from 3–6 independent experiments. scr: scramble control, vec: vector control.

Previously, we have shown that eIF3a played an important role in regulating the translation of a subset of mRNAs including p27^Kip1^ and XPC [5, 6]. We thus proposed that eIF3a might also regulate the expression of these two proteins in ovarian cancer cells. To test this possibility, we examined whether altering eIF3a level affects their expression using both A2780 cells with eIF3a knockdown and A2780/DDP cells with eIF3a overexpression. As shown in Figure 4, the protein level of p27^Kip1^ and XPC were all decreased in A2780/DDP cells with eIF3 overexpression compared with vector control, whereas the expression of these two proteins in A2780 cells with eIF3a knockdown increased compared with the scramble control.

## DISCUSSION

**Figure 5:**
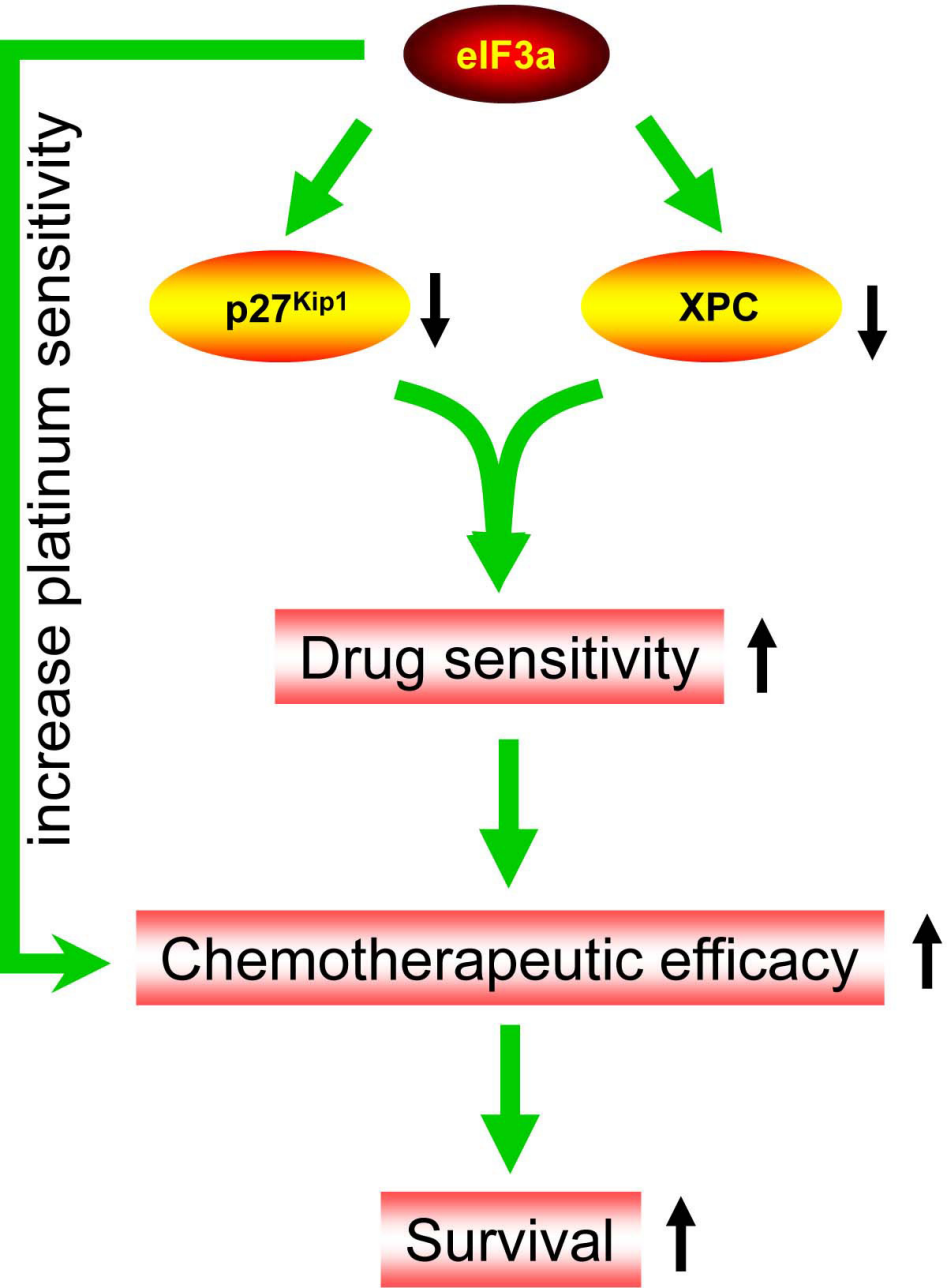
Diagram of eIF3a function in ovarian cancer eIF3a down regulates the protein level of XPC and p27^Kip1^ in ovarian cancer cells which, in turn, increases platinum sensitivity and chemotherapeutic efficacy. Thus, higher eIF3a expression patients have better survival.

In the current study, we found that eIF3a was highly expressed in DDP sensitive EOC patients compared with DDP resistance EOC patients, and higher eIF3a expression patients have better prognosis. We further showed that eIF3a increased DDP response in the EOC cells by conducting experiments of knockdown and overexpression in A2780 and A2780/DDP cells, respectively. Also, we identified that XPC and p27^Kip1^ were down regulated by eIF3a (Figure 5).

eIF3a was firstly found to be over expressed in breast cancer compared with paired normal tissues, it was thus intensively investigated in breast cancers [18]. RNA sequencing identified that eIF3a was involved in the growth and migration of breast cancer MCF-7 cells [19]. Its genetic variations (rs10787899 and rs3824830) were also reported to be strongly associated with the development of human breast cancers [15]. In addition, eIF3a was also found to be elevated in a number of other cancers, including squamous cell carcinoma of the oral cavity (OSCC), lung, cervix, esophagus, stomach and colon cancers [7]. These results indicated that eIF3a played important roles in oncogenesis and was a potential oncogene.

Our previous study further identified that eIF3a also contributed to non-small cell lung cancer (NSCLC) patients' response to platinum-based chemotherapy by regulating the expression of some DNA repair proteins [12]. However, whether eIF3a also has similar function in other cancers still remains unknown. In the current study, we demonstrated that eIF3a also correlated with DDP-based chemotherapy responses and survival of ovarian cancer patients. Patients with high eIF3a expression had both better chemosensitivity and OS rate. Based on this and our previous study, we temporarily speculate that eIF3a maybe also correlated with the chemosensitivity of platinum in patients with other cancers. Platinum is widely used to treat many cancers, however, resistance is one of the major obstacles for successful chemotherapy. Thus, predicting drug response before the initiation of chemotherapy may help to choose most proper treatment strategies for each patient and improve platinum therapeutic effect. Our result unveils that eIF3a is a possible molecular marker for the prediction and assessing its level may help design individualized treatment strategies.

In addition, we observed the association between eIF3a expression and patient survival. Based on our analysis, eIF3a was an independent factor that affected the EOC patients survival. eIF3a high expression group had higher OS and RFS rates. This result is consistent with our previous study in lung cancer patents [13]. Furthermore, another two studies also demonstrated that high eIF3a level had better survival than that with low eIF3a expression in cervical and esophageal cancer patients [20, 21]. It is interesting to note that platinum is one of the major chemotherapeutic drugs for all these three cancers. We thus further hypothesize that high eIF3a expression level in variant cancer patients may contribute to better survival after receiving platinum chemotherapy.

XPC encodes a component of the NER pathway, which is the primary repair system for removing bulky DNA lesions formed by platinum. It plays essential role in the early stages of global genome repair (GGR) NER pathway with major function of damage recognition [22]. Its polymorphisms were reported to be associated with platinum-based chemotherapy response [23]. p27^Kip1^ is an inhibitor of CDKs and plays important role in regulating cell cycle and growth arrest. It is also reported to be correlated with platinum resistance [24]. In the current study, both XPC and p27^Kip1^ was down regulated by eIF3a, which was in agreement with our previous finds in the lung cancer. This result further demonstrated that in addition to NER DNA repair pathway which was investigated previously, cell cycle also plays important role in the sensitization of eIF3a to ovarian cell response to DDP. However, the detail mechanisms and whether other cell cycle related proteins are also under eIF3a regulation require further investigation.

It should be note that the present study has several limitations. Firstly, we only investigated XPC and p27^Kip1^, however, there are some other proteins in NER DNA repair pathway also under eIF3a regulation. The role of these molecules in eIF3a regulating ovarian cancer platinum response still remains unclear. Secondly, the sample size in the current study was relative small. Thus, the correlation of eIF3a expression and ovarian cancer patients survival need to be validated in a larger sample size population. Finally, for EOC patients, the marker differentiating benign and malignant ovarian diseases is very important. However, the eIF3a expression level in the benign ovarian diseases was not reported. It is still not clear if eIF3a was such a potential marker. We will investigate this possibility in the future.

In summary, we showed that eIF3a expression level correlates with responses of ovarian cancer patients to DDP-based chemotherapy and their survival. We also showed that eIF3a may regulate the response of ovarian cancer cells to DDP via down regulating XPC and p27^Kip1^. The current study indicated that eIF3a may represent a new prognostic marker predicting DDP response and survival of EOC patients.

## MATERIALS AND METHODS

### Materials

Cisplatin, β-actin antibody and thiazolyl blue tetrazolium bromide were purchased from Sigma (St Louis, Missouri, USA). Antibodies against eIF3a, XPC and p27^kip1^ were from Santa Cruz Biotechnology (Santa Cruz, California, USA). Cell culture media and reagents were obtained from Invitrogen (Carlsbad, California, USA). All other reagents were of molecular biology grade.

### Selection of study population and acquisition of clinical information

The study protocol was approved by the Ethics Committee of Xiangya School of Medicine, Central South University. All patients were provided written informed consent in compliance with the code of ethics of the World Medical Association (Declaration of Helsinki). Eligible patients were from Hunan Provincial Tumor Hospital (Changsha, Hunan, China) or Xiangya Hospital (Changsha, Hunan, China) and diagnosed between March 2008 and March 2012. In this study, a total of 126 human ovarian cancer samples were collected after surgery. All collected tissues were embedded in paraffin after fixation in 10% formalin for histological diagnosis and IHC analysis. Among which, 46 randomly selected fresh specimens of DDP sensitive EOC samples (*n* = 23) and DDP resistance EOC samples (*n* = 23) were also collected for western blot analysis. The eligible patients for the study had to meet the following criteria: (a) histologically confirmed ovarian cancer; (b) receiving no radiotherapy and biological therapy before chemotherapy; (c) having been treated with more than 3 cycles of DDP-based chemotherapy as a first-line treatment; (d) with primary ovarian tumors; and (e) having undergone full follow-up at the hospital after treatment and been evaluated for response of chemotherapy. Patients who had the evidence of disease progression on primary treatment or within 6 months from the end of primary treatment are deemed as platinum resistant, while those had no evidence of disease progression within 6 months of the end of primary treatment are classified as platinum sensitive [25].

Exclusion criteria include: (a) pregnancy or lactation; (b) active infection; (c) symptomatic brain or leptomeningeal metastases; and (d) previous or other concomitant malignancies. All of the patients were staged according to the International Federation of Gynecology and Obstetrics (FIGO) surgical staging system [26]. All other demographic and clinical information were obtained from the 2 hospitals mentioned earlier.

### Follow-up and candidate prognostic factors

Patient follow-up was terminated on July 14, 2014. Average follow-up time was 48.32 months (median 56.0 months; range 2–60 months). OS was defined as the interval in months between EOC resection and death or the last observation. RFS was defined as the months of surgery to identification of relapse from any cause. Patients alive at the end of follow-up were censored. To determine factors influencing survival after operation, 5 conventional variables along with the expression of eIF3a were tested in all participants. All research protocols strictly complied with REMARK guidelines for reporting prognostic biomarkers in cancer [27].

### Cell culture and transfections

A2780 cell line was purchased from Institute of Biochemistry and Cell Biology, Chinese Academy of Sciences (Shanghai, China). The cells were cultured in low glucose Dulbecco's Modified Eagle Media (DMEM, GIBCO, Gaithersburg, MD, USA) and supplemented with 10% fetal bovine serum (GIBCO) at 37°C under an atmosphere of 95% air and 5% CO_2_. DDP-resistant cell line A2780/DDP derived from its parental ovarian cancer cell line A2780 by applying stepwise increases in concentrations of DDP. The A2780/DDP cells were incubated in 1 μM of DDP.

For the transfection, 2 μg DNA and 10 nM siRNA were transfected into A2780/DDP and A2780 cells in 6-well plates using Lipofectamine2000™ (Invitrogen, Carlsbad, California, USA) according to the manufacturer's instructions, respectively. After transfection for 48 h, cells were subjected to MTT assay or harvested and stored as cell pellets at −80°C until use.

### Immunohistochemistry

Tissue sections (4 μm thick) were prepared from paraffin embedded blocks. After antigen retrieval treatment in 10 mM citrate buffer (pH 6.0) at 95°C for 10 min, immunostaining was performed using the Envision System (Dako, Glostrup, Denmark) with diaminobenzidine. The tissue sections were then stained for eIF3a detection using a mouse monoclonal antibody (dilution 1:400), and a subsequently streptavidin-peroxidase system (ZSGB-BIO, Beijing, China). The negative controls for IHC were carried out under the same experimental conditions by omitting the primary antibody. The semiquantitation for intensity was scored on a scale of 0 (negative), 1 (weak), 2 (moderate) and 3 (strong) [28]. We also evaluated the approximate proportion of cells showing immunoreactive score (0 (<1%), 1 (single to 5%), 2 (6–50%), 3 (51–75%) and 4 (>75%)) to give information about the relative number of positive cells within the specimen (frequency score) [29]. These two kinds of scores were then multiplied to generate the IS for each tissue specimen. Receive operating characteristic (ROC) curve analysis was employed to assess cutoff score for overexpression of eIF3a. The score was selected as the cut-off value, which was closest to the point of maximum Youden's index (sensitivity + specificity−1) was used for determination of optimal cut-off values of the diagnostic tests. OC case designated as “negative expression” for eIF3a was those with scores below or equal to the cutoff value (IS < 2), while “positive expression” tumors were those with scores above the value (IS ≥ 2).

### MTT assay

The cell growth rate was determined by using MTT assay as we described previously [12, 30]. In briefly, cells were seeded in 96-well plates and allowed to grow for 24 hours followed by incubation with different concentrations of DDP for another 96 hours. Culture medium was then removed and cells were incubated with 1 mg/mL thiazolyl blue tetrazolium bromide for 4 hours at 37°C. The formazan was then solubilized in dimethyl sulfoxide (DMSO) and OD 570 nm was measured by a TECAN M200pro NanoQuant microplate reader (Männedorf, Switzerland). Finally, the dose-survival curves were drawn by GraphPad Prism 5.0 program (GraphPad Software, Inc.).

### Real-time RT-PCR

RT-PCR was carried out as previously described [31]. Briefly, 1 μg total RNAs were isolated by using RNeasy Mini Kit (Qiagen, Hilden, Germany) and used for reverse transcription by using primescript 1^st^ strand cDNA synthesis kit (Takara, Dalian, China) according to the manufacturers' instructions. Real-time PCR were carried out in a 7500 Real-Time PCR System (Applied Biosystems, Foster City, CA, USA) by using SYBR Premix Ex Taq (Takara) according to the manufacturer's instructions. The threshold cycle (Ct) of each reaction was determined and normalized to that of β-actin internal control.

### Western blot analysis

Total protein was extracted and separated by SDS-PAGE and then transferred onto PVDF membrane (Millipore, Bedford, Massachusetts, USA). The blocked membranes were then respectively incubated with the primary antibodies at 4°C overnight followed by HRP-conjugated secondary antibodies (KPL, Gaithersburg, Maryland, USA, 1:3000 dilution) for 1 hour at 37°C. Bands were visualized using the enhanced chemiluminescence kit (Santa Cruz Biotechnology, Santa Cruz, California, USA). The target signals were quantified by BandScan software (Bio-Rad Laboratories, Hercules, California, USA) and defined as the ratio of target protein relative to β-actin.

### Statistical analysis

Statistical analysis was performed using SPSS 17.0 software (SPSS, Chicago, Illinois, USA). Kruskall-Wallis and Mann-Whitney *U* nonparametric tests were utilized to compare differences of eIF3a expression levels. The differences of eIF3a expression level in patients were analyzed using chi-square test. The cumulative OS and RFS were evaluated using the Kaplan-Meier method and the log-rank test. Cox proportional hazards regression model was used to determine if eIF3a expression is an independent prognostic indicator. All the tests were two-sided and *p* < 0.05 was considered as statistically significant.
